# Feasibility of Conebeam CT-based online adaptive radiotherapy for neoadjuvant treatment of rectal cancer

**DOI:** 10.1186/s13014-021-01866-7

**Published:** 2021-07-23

**Authors:** Rianne de Jong, Jorrit Visser, Niek van Wieringen, Jan Wiersma, Debby Geijsen, Arjan Bel

**Affiliations:** Department of Radiation Oncology, Amsterdam UMC, University of Amsterdam, Meibergdreef 9, 1105AZ Amsterdam, The Netherlands

**Keywords:** Adaptive radiotherapy, Adaptive treatment, Rectal cancer, Conebeam CT

## Abstract

**Background:**

Online adaptive radiotherapy has the potential to reduce toxicity for patients treated for rectal cancer because smaller planning target volumes (PTV) margins around the entire clinical target volume (CTV) are required. The aim of this study is to describe the first clinical experience of a Conebeam CT (CBCT)-based online adaptive workflow for rectal cancer, evaluating timing of different steps in the workflow, plan quality, target coverage and patient compliance.

**Methods:**

Twelve consecutive patients eligible for 5 × 5 Gy pre-operative radiotherapy were treated on a ring-based linear accelerator with a multidisciplinary team present at the treatment machine for each fraction. The accelerator is operated using an integrated software platform for both treatment planning and delivery. In all directions for all CTVs a PTV margin of 5 mm was used, except for the cranial/caudal borders of the total CTV where a margin of 8 mm was applied. A reference plan was generated based on a single planning CT. After aligning the patient the online adaptive procedure started with acquisition of a CBCT. The planning CT scan was registered to the CBCT using deformable registration and a synthetic CT scan was generated. With the support of artificial intelligence, structure guided deformation and the synthetic CT scan contours were adapted by the system to match the anatomy on the CBCT. If necessary, these contours were adjusted before a new plan was generated. A second and third CBCT were acquired to validate the new plan with respect to CTV coverage just before and after treatment delivery, respectively. Treatment was delivered using volumetric modulated arc treatment (VMAT). All steps in this process were defined and timed.

**Results:**

On average the timeslot needed at the treatment machine was 34 min. The process of acquiring a CBCT, evaluating and adjusting the contours, creating the new plan and verifying the CTV on the CBCT scan took on average 20 min. Including delivery and post treatment verification this was 26 min. Manual adjustments of the target volumes were necessary in 50% of fractions. Plan quality, target coverage and patient compliance were excellent.

**Conclusions:**

First clinical experience with CBCT-based online adaptive radiotherapy shows it is feasible for rectal cancer.

*Trial registration* Medical Research Involving Human Subjects Act (WMO) does not apply to this study and was retrospectively approved by the Medical Ethics review Committee of the Academic Medical Center (W21_087 # 21.097; Amsterdam University Medical Centers, Location Academic Medical Center, Amsterdam, The Netherlands).

**Supplementary Information:**

The online version contains supplementary material available at 10.1186/s13014-021-01866-7.

## Background

Radiotherapy is standard of care in neoadjuvant treatment of intermediate and locally advanced rectal cancer, generally followed by Total Mesorectal Excision (TME) surgery [1]. In intermediate risk tumors radiotherapy is applied with a prescribed dose of 5 × 5 Gy in order to sterilize surgery planes. For locally advanced rectal cancer a dose of 25 × 2 Gy is given for downstaging to improve rates of complete resection. Radiotherapy comes inevitably at the cost of toxicity as a result of dose to healthy tissue, in case of rectal cancer mostly due to dose to bladder and small bowel [2, 3].

To ensure target coverage during treatment the radiotherapy target volume as defined on a single reference CT is enlarged with a PTV margin. The margin size is based on all uncertainties in the treatment chain of radiotherapy [4]. For rectal cancer the daily change in volume and shape is the main contributor to the PTV margin. It results in a required margin of up to 25 mm at the ventral side of the upper mesorectum [5–7] in order for the CTV to receive at least 95% of the prescribed dose for 90% of the patients.

Multiple steps have been taken in the last two decades to minimize toxicity of radiotherapy treatment by reducing dose to the healthy tissue without compromising coverage of the target volume. First, treatment delivery and planning technique evolved to intensity modulated radiation therapy (IMRT) and volumetric modulated arc therapy (VMAT), which enabled tightly shaped dose distribution around the PTV with a steep dose gradient [8–10]. Secondly, integrated in-room 3D-kV imaging (Conebeam CT (CBCT)) made it possible to position the patient and assess target coverage on a daily basis [11]. In case of insufficient target coverage due to systematic local anatomical changes or more global anatomical changes that would result in a suboptimal dose distribution, the plan could be adapted in an offline setting using repeat CT scan [12]. Online CBCT also allows a plan selection strategy where a-priori generated plans are available and the plan that best fits the anatomy of the day is selected [13–15]. We previously demonstrated that this plan selection strategy can reduce dose to the organs at risk (OAR) significantly for *individual patients for whom the population based margins (PTV = 20 to 25 mm) are too large. For the group of patients as a whole the benefit of plan selection is limited* [16].

Recently, technologies have become available that enable online adaptive radiotherapy (ART) such as in-room online MRI guidance. It combines improved image quality (with respect to CBCT) with software programs that allow automatic identification of the target volume and organs at risk and automatic re-planning [17, 18]. In a previous study we showed that there is a large dosimetric advantage of online ART for rectal cancer [19].

Currently, also a more economic online adaptive system has become commercially available that uses not MRI imaging but CBCT scans for the online workflow and combines it with artificial intelligence to support the online workflow. The goal of this study was to describe and evaluate the feasibility of such a CBCT-based online ART procedure. We selected rectal cancer (in the neoadjuvant setting) as it has large interfractional shape changes making it a perfect candidate for online ART.

This study describes and evaluates our first experience with a CBCT-based online adaptive workflow with respect to timing of different steps in the process, plan quality, target coverage and patient compliance.

## Methods

### Patients

Twelve consecutive rectal cancer patients eligible for a prescribed dose of 5 × 5 Gy according to Dutch guidelines [20] were scheduled for external beam radiotherapy (Ethos, Varian Medical Systems, Palo Alto, USA) between June 2020 and February 2021. Exclusion criteria were hip prosthesis on both sides and an inability to lie still at the treatment table for a period of 30 min. Patient compliance with respect to bladder filling and total treatment time were monitored.

### Reference CT and target volumes

A reference CT was acquired with a drinking instruction aiming at a comfortably filled bladder. To achieve this, patients were asked to void the bladder 1.5 h before the scheduled CT appointment and subsequently drink 0.5 L of fluid. All patients were positioned supine using a knee support and with arms raised over the head using a thorax support (Thorax support, MacroMedics). All patients received intra venous contrast and female patients with distal tumors vaginal contrast as well.

**Fig. 1:**
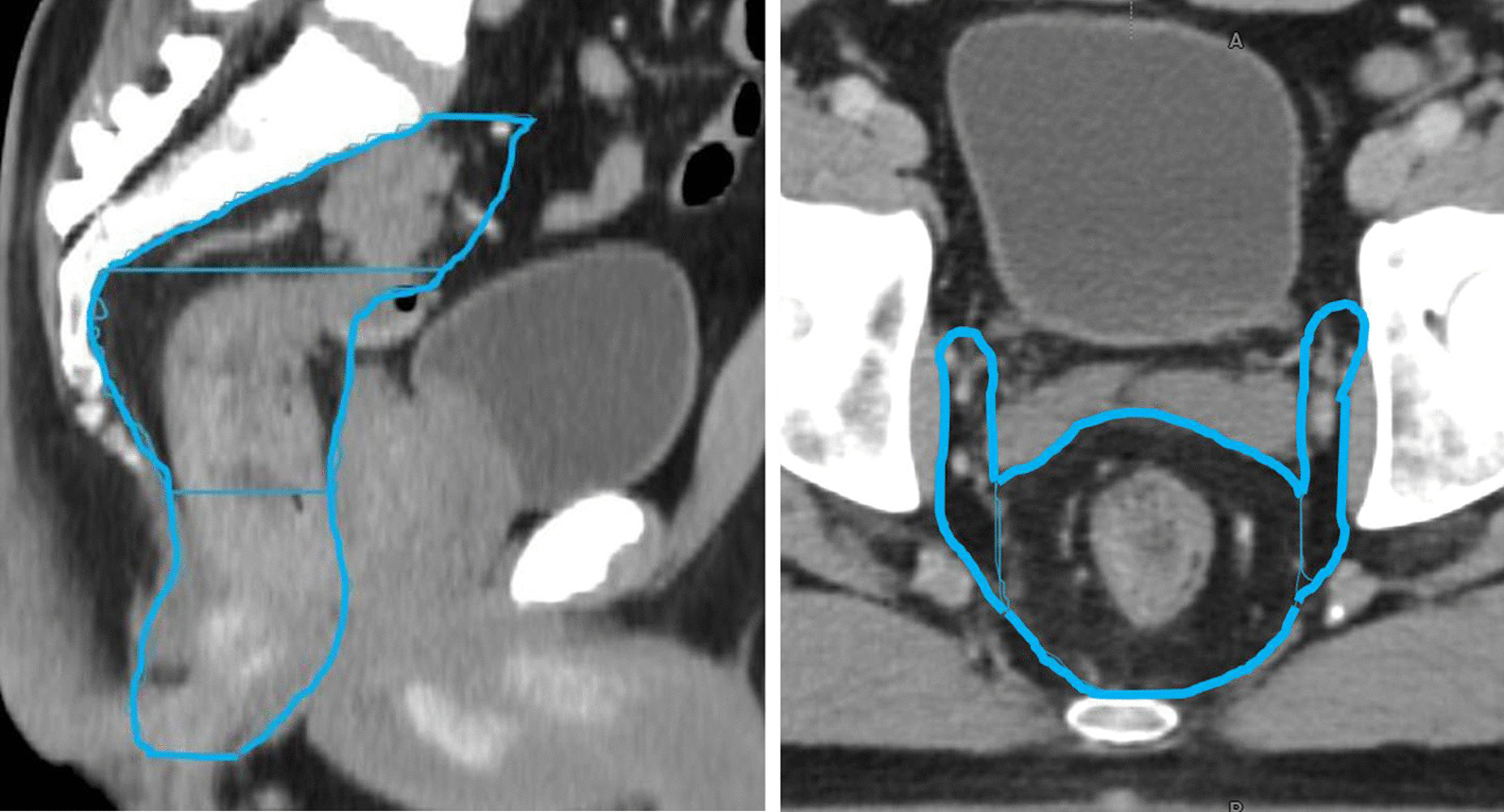
Illustration of planning CT with target definition in blue. On the right, the axial slice shows the CTV of the upper mesorectum and lymph node region left and right while on left the sagittal slice shows the lower mesorectum, upper mesorectum and presacral space (bottom to top)

Clinical target volumes were delineated (Velocity 4.1, Varian Medical Systems) using the national delineation guidelines following Valentini et al. (Fig. 1) [21]. Next to the GTV (tumor and positive lymph nodes) the radiation oncologist separately delineated the CTV upper and lower mesorectum (divided at the base of bladder), presacral space and left and right elective lymph node regions. Organs at risks (OAR) were delineated by Radiation Therapists (RTTs) according to RTOG guidelines as well as the rectum. Target volumes were reviewed by a second expert radiation oncologist.

### Margins

In all directions for all CTVs a PTV margin of 5 mm was used, with the exception in the cranial direction of the presacral, mesorectum upper and lymph node CTV and for the caudal direction of the mesorectum lower CTV. Because it was expected to be difficult to discern the cranial and caudal borders of the target volumes on CBCT it was decided to use a PTV margin of 8 mm in these cases.

### Treatment planning

The planning CT and delineations were exported from Velocity to Aria (Varian Medical System, version 16.00.00). In Ethos Treatment Management (Varian Medical Systems, version 02.00.10) a template was loaded with a departmental prioritized list of clinical goals. These clinical goals consisted of the evaluation objectives that were used for plan evaluation, as specified in the clinical protocol. Additionally, in order to achieve a more desirable dose distribution than achieved by only supplying the evaluation objectives, also optimization objectives were added as clinical goals in the template. The CT was imported into Ethos from Aria, after which the body contour was automatically delineated as well as the bony structures. If present, gas pockets and regions with contrast fluid were delineated and a material assignment was applied, where water was used as material. Next a preview of the dose distribution was automatically generated by the system. This dose preview was generated using a fluence optimized nine beams IMRT plan (system assigned) with the provided clinical goals as input. Based on the dose preview the clinical goals that were used as optimization objectives were adjusted to further improve the plan as routine practice for all patients. Subsequently, the final clinical goals and material assignments were used by the system to generate multiple deliverable IMRT and VMAT plans with fixed beam setup. From these plans the best plan deemed by the radiation oncologist was selected as reference plan and approved. As part of the QA protocol, an independent dose calculation was performed (Mobius3D 3.1, Varian Medical Systems) on the reference plan. The pass rate was required to be larger than 90%, which indicated the percentage of voxels with gamma < 1 (3%/3 mm, where the percentage was relative to the maximum dose and only voxels with a dose of more than 10% of the maximum dose were taken into account). In addition, the reference plan was delivered to a phantom (Octavius 4D, PTW, Germany) and a pass rate of at least 90% was required (gamma 3%/3 mm, where the percentage was relative to the local dose and only voxels with more than 10% of the maximum dose were taken into account).

### Adaptive workflow

**Fig. 2:**
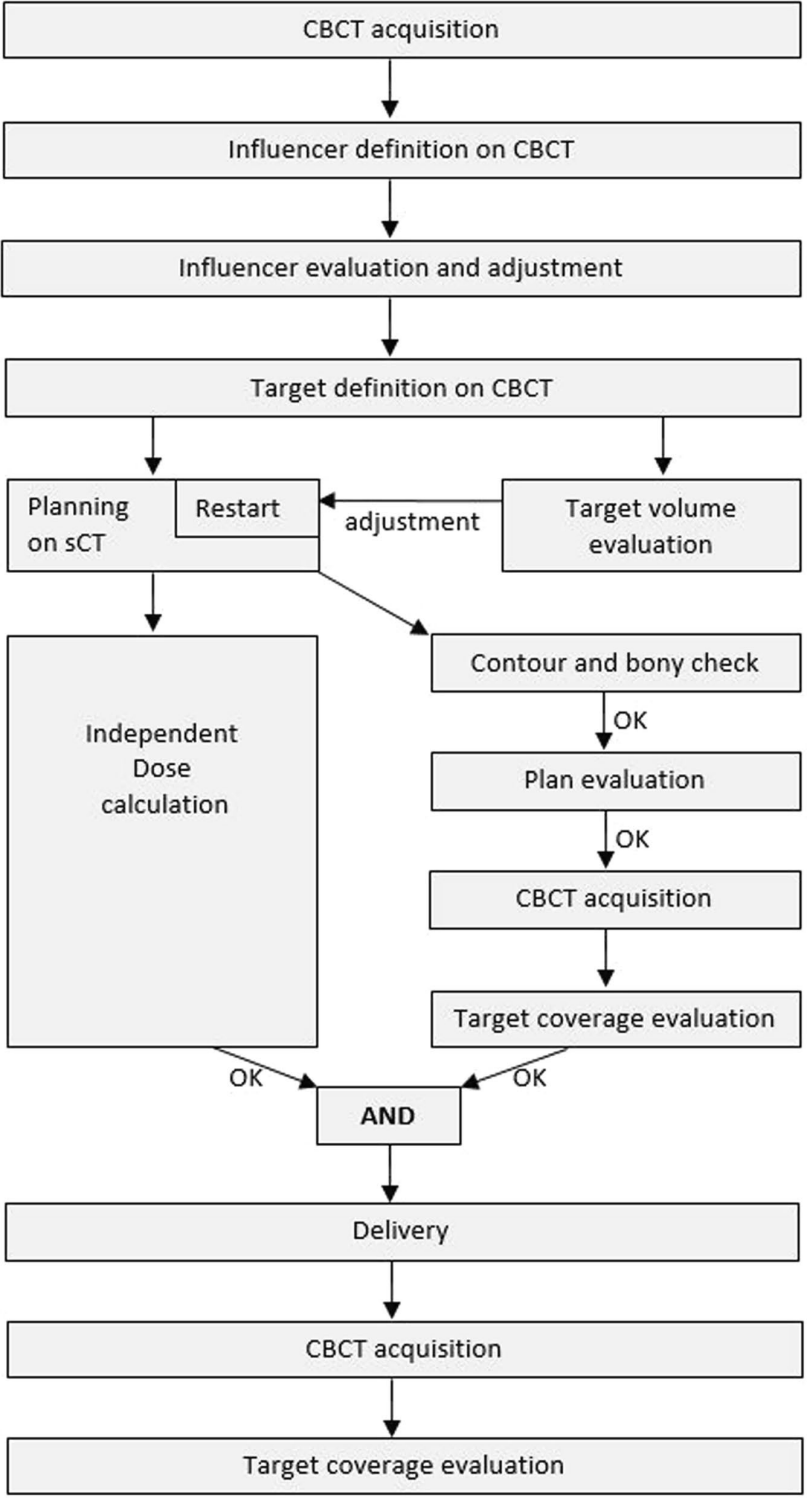
Flowchart of the *standard* online adaptive workflow

For all patients and all fractions one physicist, one radiation oncologist and two RTTs were present at the treatment machine. RTTs were in charge of running the software and adjusted structures under supervision of the radiation oncologist. For every patient, prior to the first treatment session, a 30-min timeslot was scheduled to discuss patient specific clinical target volumes and reference plan to avoid discussions during the online adaptive workflow. Because both radiation oncologist and two RTTs were evaluating contours on a single monitor a checklist was developed to streamline and order this process (Additional file 1). A flowchart of the *standard* adaptive workflow *as provided by the system* on the treatment machine is *shown* in Fig. 2.

After patient setup the first CBCT was acquired (scatter grid, 125 kV, 1080 mAs, iterative reconstruction, extended FOV if the CTV in CC direction exceeds 24 cm with a maximum of 36 cm, matrix 512 × 512).

The system generated a synthetic CT by deforming the pretreatment planning CT to the CBCT using mutual information. The resulting vector field was used by the system to propagate the body contour, bony structures, and material override structures from the pretreatment planning CT to the synthetic CT.

Subsequently, the system generated delineations using Artificial Intelligence of the following pelvic organs: rectum and bladder. In this system these structures are called ‘influencer structures’ as they influence the deformation of the target volumes using structure-guided deformable registration. If necessary, these delineations were adjusted by the RTT.

In the following step the system combined the deformation vector field and the influencer structures to automatically propagate the target volumes from the pretreatment planning CT to the CBCT. At the moment the system determined the target volumes of the patients current anatomy it presented these target structures to the users and at the same time started (1) generating a newly optimized treatment plan using the beam setup and clinical goals of the reference plan and (2) calculating the dose distribution of the unaltered reference plan but using a isocenter translation aligning the target volumes on pretreatment planning CT and those propagated to the CBCT. In both calculations the system used the patients anatomy as represented by the synthetic CT including body contour and material assignments. The newly created plan was called the adapted plan, whereas the reference plan recalculated on current anatomy was referred to as the scheduled plan.

The RTT and radiation oncologist review the propagated contours and if necessary the RTT applies adjustments. Making adjustments yields a restart of (1) and (2) described above, at the moment the adjusted target volumes were approved.

RTTs, together with the radiation oncologist and physicist, evaluated both plans by comparing the clinical goals and the dose distribution, after which the best plan was selected. After approval of the selected treatment plan a second CBCT scan (same variables as first CBCT except for 540 mAs) was acquired to verify if the target volumes were still within the PTV. The second CBCT scan was registered to the first CBCT scan using bony anatomy. If the correction was more than 1 mm in any direction a table displacement was applied. For the visual assessment if the target volume was still within PTV the system propagated the PTV structure of the adapted treatment plan on to the CBCT.

Concurrently, an independent calculation of the dose distribution was done as part of our QA protocol (Mobius, Varian Medical Systems), where a pass rate of 90% was required (gamma 3%/3 mm). Additionally, as a sanity check, the number of monitor units (MU) of the selected plan was compared to the number of MU of the reference plan.

After treatment delivery a third, post RT CBCT scan was acquired to again check if the target volume, as visible on the post treatment CBCT scan was inside the PTV of the plan used for treatment.

Timing of each step was captured to provide an overview as well as the number of fractions the target volumes needed to be adjusted by the users. Since this was a novel procedure for our department with no extensive clinical experience, unplanned events were recorded.

## Results

An overview of the twelve patients included in this study can be found in Table 1, a summary of the timing of all steps of the online adaptive workflow can be found in Table 2.

**Table 1:** Patient characteristics

	Age	Sex	Tumor stage	GTV location	Chemo	Surgery	Remarks
1	66	F	cT3bN1M0 MRF-	Mid	No	Yes	
2	82	M	T4N0M0 MRF + EMVI +	Mid	No	Yes	
3	39	M	T3N2M1	Distal	Yes	No	Reirradiation
4	62	M	cT3cN2M1 MRF +	Distal	Yes	Yes	
5	49	M	cT4bN2M1 MRF +	Distal	Yes	Yes	
6	46	F	cT4aN2bM1 MRF +	Distal	Yes	No	
7	50	M	cT3c-T4M1 MRF + EMVI +	Distal	Yes		Surgery pending chemo
8	81	M	iT3aN0Mx MRF- EMVI-	Distal	Yes		+ Oesophagus tumor, Surgery pending chemo
9	75	F	cT4N1M0 MRF +	Distal	Yes	Yes	+ Sigmoid tumor
10	47	M	cT3bN2M0 MRF-	Distal	Yes	Yes	
11	69	M	cT3N1M0 MRF- EMVI +	Proximal	No	Yes	
12	62	M	cT3bN1M0 MRF-EMVI-	Distal	No	Yes

**Table 2:** Overview of steps and duration

Time (minutes) needed for		AVG	Min	Max
1	Evaluation and adjustment system generated contours (Influencers)	4	1	11
2	Evaluation target volume without adjustments	4	2	11
3	Evaluation and adjustment target volume	9	4	21
4	Adaptive procedure before delivery (CBCT2-CBCT1)	20	11	40
5	Plan calculation	8	6	11
6	Treatment delivery	4	3	6
7	On table (CBCT3-CBCT1)	26	16	46
8	Patient entering and leaving treatment room	34	20	54

### In-room time, on-table time and patient compliance

The average total treatment time defined as the patient entering and exiting the treatment room was 34 min.

The complete online adaptive workflow including all CBCT imaging, adjustments of contours, plan calculation and treatment delivery (excluding patient alignment) took on average 26 min.

No fractions were interrupted as all patients tolerated the time needed for the procedure.

### Contour adjustments

The average time spent to evaluate and adjust the system generated contours of bladder and rectum (influencers) needed for contour propagation of the target volumes was 4 min.

Subsequently, the time spent evaluating the target volumes when adjustments were not applied was on average 4 min. If the target volumes were adjusted the average time increased to 9 min. In 30 out of 60 fractions (50%) the target volumes were adjusted after visual inspection.

### Plan calculation, plan quality and independent dose calculation

When selecting the most suitable reference plan a VMAT delivery technique was preferred to limit delivery time to minimize the possibility of intra fraction motion. Calculating the scheduled and adapted plan during the online procedure took on average 8 min (synchronous calculation).

**Fig. 3:**
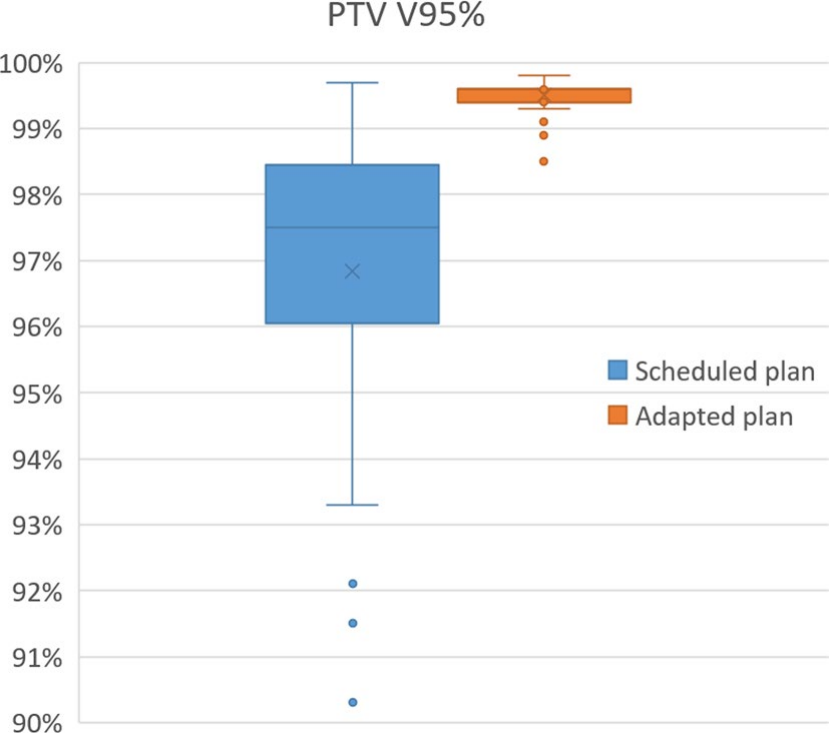
Boxplot showing PTV V95% of the scheduled and the adapted plan

**Fig. 4:**
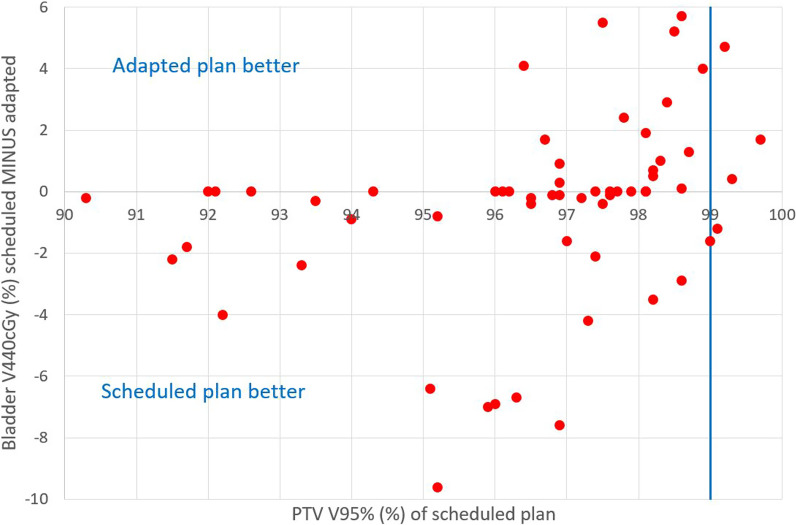
Difference between the bladder V440cGy of the scheduled and adapted plan in relation to the PTV V95% of the scheduled plan for all fractions of all patients patients (*one dot corresponds to one fraction*). The required value of 99% for the PTV V95% is indicated by the vertical blue line

To assess the quality of the scheduled and the adapted plans, the volume of the PTV receiving 95% of the prescribed dose or more (V95%) and the conformity of the 95% isodose to the PTV were checked and used as criteria for selecting the best plan. For all fractions the adapted plan was selected. For 55 out of 60 fractions V95% of PTV was less than the required 99% for the scheduled plan, while for the adapted plan this was the case for 3 fractions (Fig. 3). For fractions where the bladder V440cGy was lower for the scheduled plan than for the adapted plan, the PTV coverage of the scheduled plan was insufficient, except for two fractions (Fig. 4).

The number of MU of the selected plan never deviated more than 15% from the number of MU of the reference plan.

All plans passed the independent dose calculation.

### Online procedure

Completing the online adaptive procedure, i.e. from the first CBCT followed by contour adjustments (influencers and target volumes), plan calculation up to and including the second CBCT for verification took on average 20 min.

### Treatment delivery and intra fraction motion

Treatment delivery took on average 4 min.

For all post RT CBCTs the visual check showed no target volume outside the PTV.

### Unplanned events

**Fig. 5:**
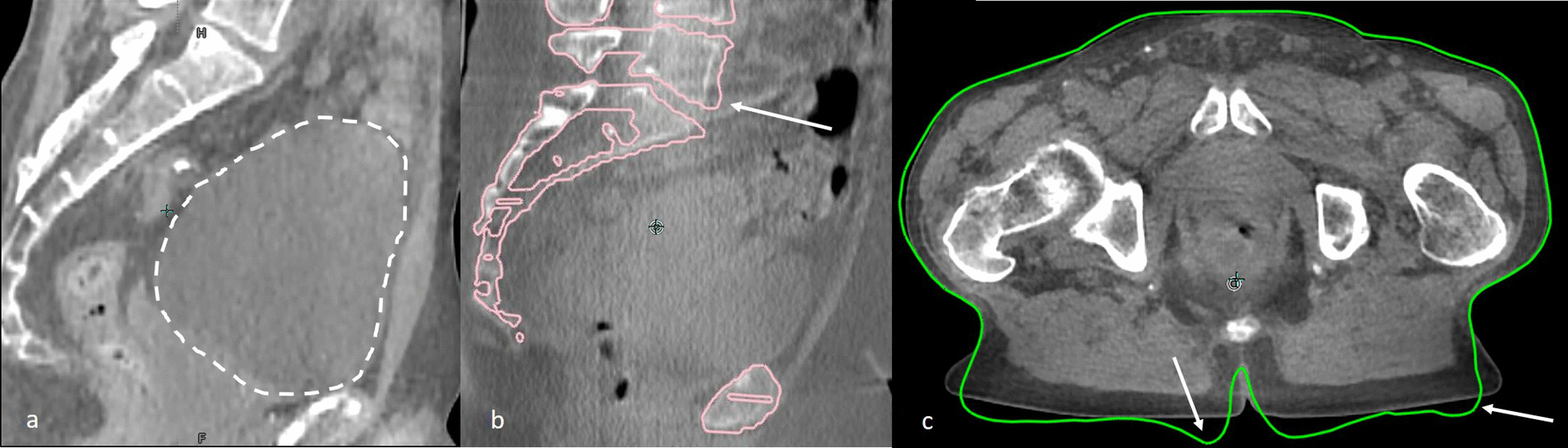
CBCT with a too full bladder at start of treatment (**a**). A small error with respect to deformable bony anatomy registration of CT to CBCT. In pink the representation of the bony anatomy of the sCT with overlay on the CBCT (**b**). A small error with respect to deformed body contour of CT to CBCT. In green the representation of the body contour of the sCT with overlay on the CBCT (**c**)

For a few fractions the adaptive procedure did not go according to plan as a result of the following:

1. the workflow was interrupted after first CBCT due to a very full bladder in one fraction, because it was expected that the patient required voiding the bladder before the end of the fraction. (Fig. 5a).
2. the workflow was completely restarted after the second CBCT because of insufficient target coverage due to intra fraction motion of a large gas pocket in one fraction.
3. after the second CBCT there was insufficient target coverage due to limited intra fraction motion of a gas pocket. Coverage was restored with a table shift in one fraction.
4. for one patient the synthetic CT had a small error in 3 out of 5 fractions with respect to bony anatomy registration that resulted in a need to extensively adapt the presacral delineation in that region (Fig. 5b).
5. for one patient the synthetic CT had an error in all fractions with respect to body contour definition (Fig. 5c). This error was ignored because limited impact on the dose distribution was expected.

## Discussion

To our knowledge, this is the first study that describes an online CBCT-based adaptive radiotherapy workflow for rectal cancer in the neoadjuvant setting and reports the first clinical experience. All scheduled patients were treated as intended. The workflow worked well but was labor intensive as it required a multi-disciplinary team at the treatment machine and compared to our previously used plan selection protocol [14, 16] time slots at the treatment machine are prolonged (15 min vs 35 min). Patient compliance was not affected.

### Treatment times

Intven et al. [22] was the first to report daily adaptive radiotherapy for rectal cancer patients treated with 5 × 5 Gy and similar target volumes and showed it was a feasible strategy for MR guided radiotherapy.

Overall, the online adaptive workflow they described took longer in comparison to our workflow. Median of the total treatment time defined as the time between first MR scan and end of treatment delivery was longer with a median of 43 min (IQR 9 min), as compared to 26 min (range 16–46) in our study. They report contouring time with a median of 13 min (IQR 11). In our workflow contours can be evaluated and adapted in two steps: (1) the system generated structures of the pelvic organs (the influencers), i.e. rectum and bladder, and (2) the target volumes. This first step is not part of the MR guided workflow. When only the system generated delineations of bladder and rectum were evaluated and/or adjusted it took on average 4 min. If on top of that the target volumes needed to be adapted this increased to an average of 9 min.

When selecting a reference plan for our online workflow the VMAT technique was favored for its fast delivery over IMRT. The 5-field IMRT technique used for delivery by Intven et al. [22] resulted in a median of 7 min (IQR 1) treatment delivery time compared to an average of 4 min (range 3–6) in our study.

### Imaging

The system provided the possibility of iterative reconstructed CBCT scans and produced sufficient image quality for the evaluation and adjustment of the influencer structures and target volumes. Incidental unfavorable patient anatomy, causing a lot of streak artefacts (moving gas in small bowel), increased the time needed to evaluate and adapt contours.

Online adaptive radiotherapy is to date commonly reported using an MR-Linac. MR guided radiotherapy has the potential benefit of better soft tissue contrast compared to CBCT scans [22]. Possibly, the MRI image quality could result in more accurate re-delineation of contours which needs to be investigated. MRI guided imaging could also enable the visualization of the GTV and treatment response which is not possible using CBCT-based online adaptive radiotherapy.

### Margins and intra fraction motion

We previously compared plan selection and online ART with respect to dose to the healthy tissue [19]. In that study a 3 mm PTV margin around the total target volume was used. As this was our first clinically implemented online adaptive workflow with the Ethos system we decided to start with a slightly larger margin: 5 mm in all directions with the exception of 8 mm in cranial direction for upper mesorectum, presacral space and elective lymph node regions and 8 mm in caudal direction for lower mesorectum because it was expected to be difficult to discern the cranial and caudal border of the target volume on CBCT. In this decision it was also taken into account that when using smaller margins possibly more adjustments of small deviations would be needed, consequently increasing the time needed for the online ART process. Conversely, larger margins would possibly mean less adjustments are needed of small deviations, but this would yield less advantage of the online ART process with respect to dose to the OARs. Due to the different margins, the results in terms of dosimetric effect of online adaptive ART of the present paper are not directly comparable with e.g. the procedure with the plan-of-the-day [16].

To assess target coverage in the context of intra fraction motion, we also acquired a pretreatment and post treatment CBCT scan. As stated, the adapted plan needed to be shifted in one fraction and workflow was interrupted in one fraction because of insufficient target coverage. Post treatment CBCT scans showed the target volume was within PTV for all performed adapted treatments. Whether margins can be reduced further needs to be investigated. Intven et al. [22] started using 10 mm PTV margin around the mesorectum in all directions and 8 mm PTV margin around lymph node regions. After 25 patients they reduced margins to the mesorectum to 4 mm in all directions except for 6 mm in CC and ventral direction. For the lymph node regions the margins were reduced to 4 mm in all directions except for 6 mm in CC direction. These PTV margin reductions were based on an evaluation of adequate coverage to the target on the post treatment MRI scans.

### Resources

When designing the adaptive protocol we aimed for an RTT led workflow from the start. Therefore, we protocolized that RTTs would drive the software under supervision of the radiation oncologist and the medical physicist for the first 12 patients. RTTs were already experienced in CBCT-based online IGRT and plan selection for rectal cancer. For the adaptive workflow, additional training for the RTTs was provided in the form of the ESTRO Falcon delineation course followed by a target definition workshop for departmental specific criteria. This was combined with 40 h of individual training on a research version of the clinical software after individual instructions. A total of 5 RTTs were trained. To streamline and order the process of evaluation and adaptation of contours we developed a checklist, see Additional file 1. An offline QA protocol would help and is currently under investigation as well as the possibility to remotely view the screens to support a workflow without the physician and/or physicist physically present at the treatment machine.

Intven et al. [22] report that their workflow started with recontouring performed by radiation oncologists for the first 12 patients. As of patient 13 RTTs were trained followed by a gradual shift in the responsibility of the recontouring to RTTs. This shift towards an RTT led online adaptive procedure is in line with the results of an international survey published by McNair et al. [23].

In our workflow also the medical physicist was present at the treatment machine to approve the adapted plan and to evaluate the independent dose calculation. A traffic light protocol would further enable an autonomous RTT led workflow in the future as is described by Betgen et al. [24].

The time slots at the treatment machine were 40 min. The difference between the on table time (26 min) and the total treatment time as defined by patient entering and leaving the treatment room (34 min) is large. This is most likely the result of a not fully booked patient schedule, leaving extra time for social interaction between RTTs and the patients.

### Patient compliance

In general, rectal cancer patients receive a bladder filling instruction that aims at comfortable full bladders during treatment. We estimated the treatment time for online ART to be about 30 min and did not change drinking instructions as a (comfortable) full bladder is beneficial for dose to the bladder and it improves CBCT image quality. The average in-room time as defined by patient entering and leaving the treatment room was on average 34 min but with outliers up to 54 min. All patients were compliant with these treatment times, although for one fraction the workflow was interrupted based on an extremely full bladder on the first CBCT scan.

Prolonged treatment times (compared to previous plan selection time slots) can affect patient comfort as they are immobilized with the arms elevated. As a result we needed to alter arm position to arms crossed on the chest for one patient after the first treatment fraction.

### Scheduled and adapted plan

The system automatically provides a scheduled plan next to the adapted plan. The user is able to compare both plans with respect to clinical goals and dose distribution. In this study the scheduled plan was never selected (Fig. 4).

### Future directions

With a feasible CBCT-based online adaptive workflow a new resource has become available for radiotherapy departments to improve treatment. Whether the CBCT image quality suffices for other treatment sites than rectal cancer needs to be investigated.

To improve CBCT-based online ART for rectal cancer effort has to be made to assess remaining and new uncertainties to calculate optimal margins and evaluate whether using online adaptive radiotherapy will translate into a clinically relevant reduction of toxicity and improvement of patient’s quality of life.

Also the supporting software tools in this process needs to be optimized to reduce time per fraction.

## Conclusion

CBCT-based online adaptive radiotherapy in the neo adjuvant treatment of intermediate and locally advanced rectal cancer is feasible and appears to be a promising strategy to reduce PTV margins and thus toxicity.

## Supplementary Information

**Additional file 1**. Check list online adaptive radiotherapy used to manage and order the steps in de workflow.

## Data Availability

The datasets generated and/or analyzed during the current study are not publicly available since the participants did not consent in sharing the data with third parties.

## Abbreviations

ART: Adaptive radiation therapy
CBCT: Conebeam CT
CT: Computed tomography
CTV: Clinical target volume
GTV: Gross tumor volume
Gy: Gray
IMRT: Intensity modulated radiation therapy
MU: Monitor unit
OAR: Organs at risk
PTV: Planning target volume
RTT: Radiation therapist
VMAT: Volumetric modulated arc therapy
