# Supplementary material for: Feasibility of Conebeam CT-based online adaptive radiotherapy for neoadjuvant treatment of rectal cancer
Source: Radiat Oncol. 2021 Jul 23;16:136. doi: 10.1186/s13014-021-01866-7 (PMC8305875; doi:10.1186/s13014-021-01866-7)
Supplement: Supplementary file 1 — Additional file 1. Check list online adaptive radiotherapy used to manage and order the steps in de workflow. [file 13014_2021_1866_MOESM1_ESM.docx]

Check form online adaptive workflow – Rectum

Influencer Review: RTT 1/2

⃝ Bladder

⃝ Rectum

Target Review: RTT 1/2 + RO

Sagital viewer: ⃝ Caudal border lower mesorectum

⃝ Cranial border upper mesorectum

⃝ Cranial border presacral space

Axial viewer: C*ranial to caudal*

⃝ Lateral and dorsal borders

C*audal to cranial*

⃝ Ventral borders

Plan Review: RTT + RO + Physicist

⃝ Body & bones (sCT)

⃝ Isodose 95%-105%

⃝ Goals

⃝ MU check

⃝ CB2: target coverage

Mobius: Physicist

⃝ Approvals

⃝ Details gamma

⃝ Orthogonal images sCT

⃝ CB3: target coverage
